# The *In Vitro* Adsorption Ability of *Lactobacillus acidophilus* NCFM to Benzo(a)pyrene in PM_2.5_

**DOI:** 10.1155/2021/6290524

**Published:** 2021-01-07

**Authors:** Lili Fu, Yan Ning, Hongfei Zhao, Junfeng Fan, Bolin Zhang

**Affiliations:** ^1^College of Biological Science & Biotechnology, Beijing Forestry University, Beijing 100083, China; ^2^Beijing Key Laboratory of Forest Food Processing and Safety, Beijing 100083, China

## Abstract

The objective of this work was to explore the ability of lactic acid bacteria strains to bind benzo(a)pyrene (B(a)P) existing in PM_2.5_. In this study, we examined the ability of *Lactobacillus acidophilus* NCFM to bind B(a)P in the simulated PM_2.5_ environment. Among the tested 5 strains, *Lactobacillus acidophilus* NCFM exhibited the best capacity to bind B(a)P, and its B(a)P binding percentage was 60.00%. Simulations of organic and inorganic systems which represent PM_2.5_ indicated that B(a)P could be absorbed by strain *L. acidophilus* NCFM. For the inorganic system of pH 5, *L. acidophilus* NCFM bound 92.74% B(a)P with a cell concentration of 1 × 10^10^ cfu/mL at 37°C for 8 hr. Regarding the organic system with pH 6, 73.00% B(a)P was bound by strain *L. acidophilus* NCFM after this bacterium was incubated at 37°C for 10 min. A quick B(a)P binding by this probiotic bacterium took place in the organic system. The removal of B(a)P from PM_2.5_ was significantly related to incubation time, cultivation temperature, pH, and cell concentration. Thus, our finding shows that long-term consumption of *L. acidophilus* NCFM is beneficial for the reduction of B(a)P towards the population who are exposed to PM_2.5_, although the ability of this bacterium to adsorb B(a)P is partly affected by the differences in the origin of PM_2.5_.

## 1. Introduction

Particulate matter, harmful suspended particles existing in air environment, is referred as small particles less than 10 micrometers. The particulate matter is easily deposited in the upper respiratory tract and causes toxicity to the human body. Such particulate matters which are less than 2.5 micrometers in diameter, i.e., PM_2.5_, are more dangerous and have a direct damage to lung health due to long-term contact of bronchioles and alveoli [1]. The smaller the particle diameter is, the deeper into the respiratory tract happens. Meanwhile, toxicological data show that particulate matters not only affect the respiratory ability but also are harmful to cardiovascular, nervous, and immune functions [2]. Thus, the World Health Organization (WHO) defines particulate matter as a class of carcinogens and addresses that different degrees of physiological and pathological changes will take place for the particulate matter-carrying population [3].

Generally, PM_2.5_ consists of sulfate, nitrate, ammonium salts, carbonaceous particles, metal particles, minerals, and polycyclic aromatic hydrocarbon (PAH) family [4, 5]. Benzo(a)pyrene (B(a)P), a member of the family PAH, is usually found in its PM_2.5_. The presence of B(a)P in PM_2.5_ significantly strongly increases tumor cell migration and invasion, causing mutagenesis and carcinogenicity [6]. B(a)P from the PAH family is regarded as the most abundant and toxic component existing in PM_2.5_ and has long-term harm to people. Thus, it is practicable to use B(a)P as a marker to elevate the toxicity of PM_2.5_ [7].

In recent years, there has been an increasing interest in PM_2.5_ which is a significant threat to human health [8]. In addition to activated carbon and modified mesoporous organosilica which are traditionally used as adsorbing agents of PM_2.5_, microbial community is also becoming an important part of filtration media, and lactic acid bacteria strains could be explored as a bio-binding material for the removal of PM_2.5_ [9]. *Lactobacillus* strains, as food-grade microorganisms from lactic acid bacteria, have good intestinal adhesion ability of epidermal cells and thus offer various health-promoting benefits to the human body [10]. The communication between *Lactobacillus* strains defined as probiotics and intestinal cells can stop the colonization of pathogens from the intestine and enhance the immunity of the body [11]. *Lactobacillus* strains have been documented to have good adsorption to mutagenic compounds, thus being a biological agent for anticancer or antimutagenic effect [12]. Some *Lactobacillus* strains are also proved to significantly reduce the mutagenicity of B(a)P via their binding ability [13, 14]. In our cases, *L. plantarum* CICC 22135 and *L. pentosus* CICC 23163 exhibited high efficiency in removing B(a)P from the aqueous medium [15]. Moreover, such *Lactobacillus* strains showed a good B(a)P binding ability under simulated starch conditions, but their B(a)P binding percentages depended on starch concentrations [16]. Studies have shown the importance of bacteria in binding PAHs, and this reduction is significantly affected by initial PAH concentrations, bacterial population, and pH of media; especially, the highest reduction was related to B(a)P [17]. Obviously, the roles of *Lactobacillus* strains in B(a)P binding are affected by the environments in which they survive. To date, few relevant studies on whether *Lactobacilli* strains are still able to bind B(a)P in PM_2.5_ with complex and diverse particulate matter have been done. Therefore, this present study was designed to investigate the possibility of strain *L. acidophilus* NCFM to remove PM_2.5_ toxicity in terms of its B(a)P binding ability. Possible factors affecting these tested strains to bind B(a)P were also discussed when they were cultured in the simulated PM_2.5_ systems. Imaging the cell morphology of *L. acidophilus* NCFM in the simulated PM_2.5_ systems via atomic force microscopy (AFM) was presented.

## 2. Materials and Methods

### 2.1. Bacterial Strains

Several LAB strains including *Lactobacillus plantarum* 121, *Leuconostoc mesenteroides* DM1-2, *Lactobacillus acidophilus* NCFM, *Lactobacillus paralimentarius* 412, and *Lactobacillus pentosus* ML32 were obtained from the China Center of Industrial Culture Collection (CICC) and tested for this study.

### 2.2. Preparation of Bacterial Suspensions

The lyophilized preparations of five strains transferred into 5 mL de Man–Rogosa–Sharpe (MRS) medium were firstly activated at 37°C, respectively. Then, they were inoculated into the MRS medium at 37°C for 12 hr incubation with 4% inocula. Their cells were collected by centrifugation (4°C, 5000 rpm, and 10 min) and washed twice with sterile water. The cell concentrations of these tested strains were finally adjusted to 5 × 10^9^ cfu/mL prior to use.

### 2.3. Preparation of Two B(a)P Simulation Systems

#### 2.3.1. Inorganic System

The PM_2.5_-based inorganic compositions simplify the artificial inorganic system. Artificial inorganic system (1.0 mL) consisting of 100 *μ*L of B(a)P working solution (10 *μ*g/mL), 300 *μ*L of sodium sulfate solution (100 *μ*g/mL), 300 *μ*L of ammonium sulfate solution (100 *μ*g/mL), and 100 *μ*L of sterile water was designed according to Dhananjay et al. with some modifications [5].

#### 2.3.2. Organic System

Regarding the organic compositions of PM_2.5_ which belong to the family PHAs, PHA Mix (EPA 610), purchased from Accredited Chemical Testing Lab with a purity ≥98.5%, was used to simulate the organic system of B(a)P. The PHA Mix consists of 16 various concentrations of organic compounds, including benzo[k]fluoranthene of 99.37 ± 1.4 mg/L, chrysene of 99.59 ± 1.41 mg/L, phenanthrene of 98.9 ± 1.31 mg/L, benzo[b]fluoranthene of 202.3 ± 2.69 mg/L, benzo[ghi]perylene of 200.6 ± 2.67 mg/L, fluoranthene of 198.3 ± 2.64 mg/L, fluorine of 200.4 ± 2.66 mg/L, naphthalene of 1000 ± 3.74 mg/L, acenaphthene of 991 ± 6.1 mg/L, acenaphthylene of 2018 ± 12.31 mg/L, anthracene of 101.2 ± 1.34 mg/L, benzo[a]anthracene of 99.98 ± 1.41 mg/L, benzo[a]pyrene of 100.8 ± 1.34 mg/L, dibenz[a,h]anthracene of 202 ± 2.86 mg/L, indeno[1,2,3-cd]pyrene of 98.61 ± 1.39 mg/L, and pyrene of 100.6 ± 1.33 mg/L. The simulated organic system of B(a)P was composed of 100 *μ*L PHAs (100 *μ*g/mL) and 900 *μ*L sterile water.

The separation of sixteen kinds of PAHs within 35 min showed good linearity in the range of 0.10–5.00 *μ*g/mL, and the detection limit of B(a)P was 0.90 *μ*g/mL [18, 19].

### 2.4. Binding of Tested Strains to B(a)P in Two Simulated Systems

#### 2.4.1. Inorganic System

The artificial inorganic system (100 *μ*L) plus 900 *μ*L sterilized water, containing 1.0 *μ*g/mL B(a)P, was added to 1.0 mL bacterial suspension. After incubation at 37°C for 4 hr, the supernatant was collected by centrifugation (3000 r/min, 5 min). Chloroform (500 *μ*L) was added to the supernatant to produce the organic phase for the detection of B(a)P. The control was designed as 1.0 *μ*g/mL aqueous solution of B(a)P without bacterial cell addition. For each sample, B(a)P was detected by HPLC with the following conditions: UV detection of 290 nm wavelength, mobile phase of pure methanol, selection of room temperature as column temperature, flow rate of 1.0 mL/min, and injection volume of 20.0 *μ*L [18].

#### 2.4.2. Organic System

We selected 100 *μ*L PHAs plus 900 *μ*L sterilized water which were added to 1.0 mL bacterial suspension. After incubation at 37°C for 4 hr, the level of B(a)P existing in the supernatant collected by centrifugation (3000 r/min, 5 min) was detected by HPLC. The control was 1.0 *μ*g/mL PHAs (1.0 mL) without bacterial cell addition. B(a)P was UV-detected at 290 nm wavelength by HPLC. Acetonitrile and water were used as the mobile phase. Column temperature was room temperature with a flow rate of 1.0 mL/min as well as injection volume of 20.0 *μ*L. For gradient elution analysis, A was acetonitrile and B was water. 60% A plus 40% B was used for isocratic elution for 1 min. After A was elevated from 60% to 100% within 20 min, eluting with 100% A was kept for at least 22 min. Then, elution A was reduced to 60% in 8 min and equilibrated with 60% A for 10 min.

#### 2.4.3. The Binding Percentage of Strains to B(a)P

The binding percentage of the tested bacteria to B(a)P was calculated according to the following equation:

(1) $$\begin{array}{c} B_{r}=\frac{\left(B_{s}-S\right)}{B_{s}}\times 100\%, \end{array}$$

where *B*_*r*_ represents the binding rate of the tested bacterial cells to B(a)P (%); *B*_*s*_ indicates the level of B(a)P from the sample blank (*μ*g/mL); and *S* is the level of B(a)P for each supernatant harvested from the tested bacterial cultivation (*μ*g/mL).

### 2.5. Factors Affecting the Ability of *L. acidophilus* NCFM to Bind B(a)P

#### 2.5.1. Effect of Incubation Time

For either of the simulated inorganic or organic system, *L. acidophilus* NCFM was grown in both systems for the observation of its B(a)P binding ability under different incubation times at 37°C. After incubated in two simulated systems for 10 min, 60 min, 240 min, 480 min, 1080 min, and 1440 min, respectively, the percentage of strain NCFM to bind B(a)P was detected.

#### 2.5.2. Effect of Temperature

For either of the simulated inorganic or organic system, *L. acidophilus* NCFM was cultivated in the two simulated systems for the observation of its B(a)P binding ability under different incubation temperatures. The ability of *L. acidophilus* NCFM to bind B(a)P was evaluated after this strain was incubated at 4°C, 15°C, 23°C, and 37°C for 8 hr, respectively.

#### 2.5.3. Effect of pH

For either of the simulated inorganic or organic system, *L. acidophilus* NCFM was cultured in the two simulated systems for the observation of its B(a)P binding ability under different pH values at 37°C. The ability of *L. acidophilus* NCFM to bind B(a)P was evaluated after this strain was incubated at pH 3, 4, 5, 6, 7, 8, and 9 for 8 hr, respectively. The sample was adjusted to a specific pH value using a degassed phosphate buffer solution.

#### 2.5.4. Effect of Cell Concentration

For either of the simulated inorganic or organic system, *L. acidophilus* NCFM was grown in the two simulated systems for the observation of its B(a)P binding ability under cell concentrations. *L. acidophilus* NCFM was inoculated into the two simulated systems at cell concentrations of 1 × 10^8^ cfu/mL, 1 × 10^9^ cfu/mL, and 1 × 10^10^ cfu/mL, respectively. Estimation of cell concentrations during cultures was done by turbidimetry [20]. After incubated at 37°C for 8 hr, the ability of this strain to bind B(a)P was evaluated in terms of its cell concentrations.

### 2.6. The Optimal Parameters for *L. acidophilus* NCFM to Bind B(a)P

According to Section 2.5, the optimal conditions are obtained from two simulated systems. *L. acidophilus* NCFM was cultured in the optimal conditions to calculate the best binding percentages of B(a)P.

### 2.7. Analysis of Atomic Force Microscopy (AFM)

Atomic force microscopy was used to observe the possibility of *L. acidophilus* NCFM (10^9^ cfu/mL) to bind B(a)P. Four groups were prepared as follows. The first group was the inorganic system which contains 10 *μ*g/mL B(a)P and strain NCFM; the second was the organic system with 10 *μ*g/mL B(a)P and strain NCFM; the third was 10 *μ*g/mL B(a)P solution plus strain NCFM; and the fourth was only bacterial suspension as the control. These mixtures were incubated at 37°C for 1 hr and then diluted to a final concentration of 10 *μ*g/mL. 5 *μ*L was sampled and dispersed onto a mica carrier (PELCO mica disc 10 mm) for drying at room temperature. AFM images were captured by using a scanning probe microscope (NTEGRA Spectra, NT-MDT Co., Ltd., Moscow, Russia) in the tapping mode.

### 2.8. Statistical Analysis

Three replicates for each experiment were done. Statistical analyses (ANOVA) were performed using SPSS 19.0 statistical software. Results are expressed as means ± standard deviation (SD). Statistical differences are considered to be significant when *P* ≤ 0.05 takes place.

## 3. Results

### 3.1. The Ability of the Tested Strains to Bind B(a)P

**Figure 1:**
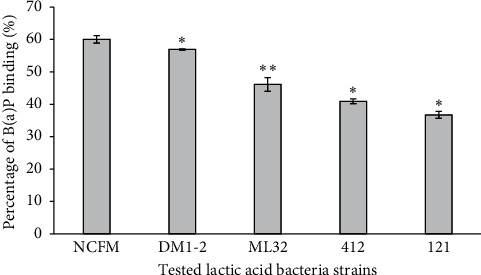
B(a)P binding percentage of lactic acid bacteria strains. Note: ^*∗*^*P* < 0.05 and ^*∗∗*^*P* < 0.01 compared with strain NCFM.

As shown in Figure 1, the five tested bacteria had different abilities to bind B(a)P. *L. acidophilus* NCFM showed the best capacity for the adsorption of B(a)P, followed by strain 121, strain ML32, strain DM1-2, and strain 412. 60.00% B(a)P was bound by *L. acidophilus* NCFM in the present study. It was seen that the ability of lactic acid bacteria to bind B(a)P was strain-dependent. To better understand the roles of various factors in determining the efficiency of B(a)P binding, only *L. acidophilus* strain NCFM was chosen for further study.

### 3.2. Factors Affecting the Ability of *L. acidophilus* NCFM to Bind B(a)P

Several factors including incubation time, incubation temperature, pH, and cell concentrations have been reported to directly affect the possibility of potential probiotic bacteria to bind B(a)P if they were presented to different media [21]. To show the possible potential of *L. acidophilus* NCFM to bind B(a)P in PM_2.5_, we designed two simulated systems, i.e., inorganic and organic systems, as imaginary PM_2.5_ to observe the B(a)P adsorbing ability of this strain was subjected to various variables.

#### 3.2.1. Incubation Time

**Figure 2:**
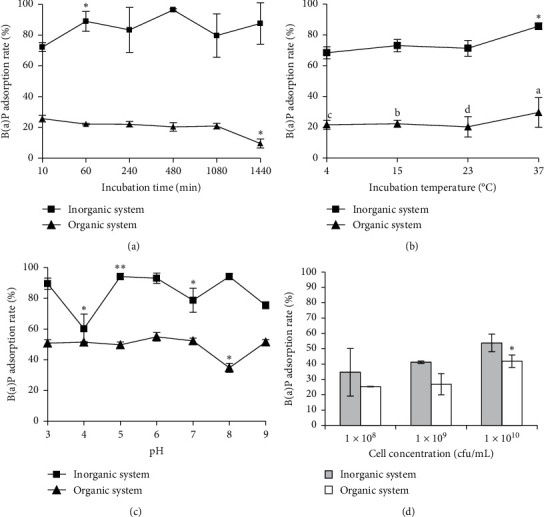
Factors affecting the binding ability of *L. acidophilus* NCFM to B(a)P existing in two simulated PM_2.5_, i.e., inorganic system and organic system: (a) effect of incubation time on B(a)P binding by strain NCFM, (b) effect of temperatures on B(a)P binding by strain NCFM, (c) effect of pH on B(a)P binding by strain NCFM, and (d) effect of cell concentrations of strain NCFM on its B(a)P binding. Note: ^*∗*^*P* < 0.05 and ^*∗∗*^*P* < 0.01 compared with the control group.

As shown in Figure 2, the highest percentage of strain NCFM to bind B(a)P in the simulated inorganic system was 96.34% when this bacterium was incubated for 8 hr. As the simulated inorganic system was concerned, no clear correlation between the B(a)P binding percentage of strain NCFM and its incubation time was observed (Figure 2(a)). For the organic system, however, the binding of *L. acidophilus* NCFM to B(a)P was a quite quick process. It only took 10 min for this bacterium to bind most B(a)P. 25.66% B(a)P was absorbed by this strain after 10 min incubation. It was seen that the binding percentage of strain NCFM to B(a)P in the inorganic system was higher than that in the organic system at the same incubation time. Thus, the component's complex from PM_2.5_ might significantly affect the adsorption of strain NCFM to B(a)P.

#### 3.2.2. Temperature

The temperature ranges from 4°C to 37°C were chosen to investigate the role of incubation temperature in affecting the ability of *L. acidophilus* NCFM to bind B(a)P in PM_2.5_ (Figure 2(b)). 85.64% B(a)P was bound by strain NCFM in the simulated inorganic system, but only 29.60% B(a)P was bound in the simulated organic system at 37°C. In both simulated systems, however, it was noted that an elevating incubation temperature was helpful for *L. acidophilus* NCFM to bind more B(a)P.

#### 3.2.3. pH

As the pH ranges from 3 to 9 were concerned, most B(a)P was bound by *L. acidophilus* NCFM when pH of both simulated systems was 5 (Figure 2(c)). Strain NCFM bound 94.11% B(a)P for the simulated inorganic system at pH 5 and 54.93% B(a)P for the simulated organic system at pH 6. An acidic to near-neutral medium surrounding seemed to be useful for *L. acidophilus* NCFM to bind more B(a)P from PM_2.5_, although this bacterium bound B(a)P more in the simulated inorganic system than in the simulated organic system.

#### 3.2.4. Strain Concentration

It is apparent that the higher the L. acidophilus NCFM cell concentrations are in both simulated systems, the more B(a)P will be bound (Figure 2(d)). About 53.75% B(a)P was absorbed by strain NCFM when its cell concentration was 1 × 10^10^ cfu/mL in the simulated inorganic system, while 41.85% B(a)P was bound in the same cell concentration.

### 3.3. The Optimal Conditions for *L. acidophilus* NCFM to Bind B(a)P

Summary of the aforementioned available data indicated that strain NCFM was able to bind B(a)P either in the inorganic or organic system. When this bacterium was incubated with a cell concentration of 1 × 10^10^ cfu/mL at 37°C for 8 hr in the inorganic system of pH 5, strain NCFM bound the highest B(a)P (92.74%). The optimal parameters for strain NCFM to bind B(a)P were 1 × 10^10^ cfu/mL cell concentration and 37°C for 10 min of incubation in the organic system of pH 6. About 73.00% B(a)P was absorbed by this strain under the optimal conditions. It was seen from our present data that at least 60.00% B(a)P could be removed in the presence of *L. acidophilus* NCFM. Obviously, *L. acidophilus* NCFM strain might have an ability to remove B(a)P from PM_2.5_. Thus, consumption of this probiotic bacterium should be beneficial for humans to reduce the damage of PM_2.5_.

### 3.4. AFM Analysis

**Figure 3:**
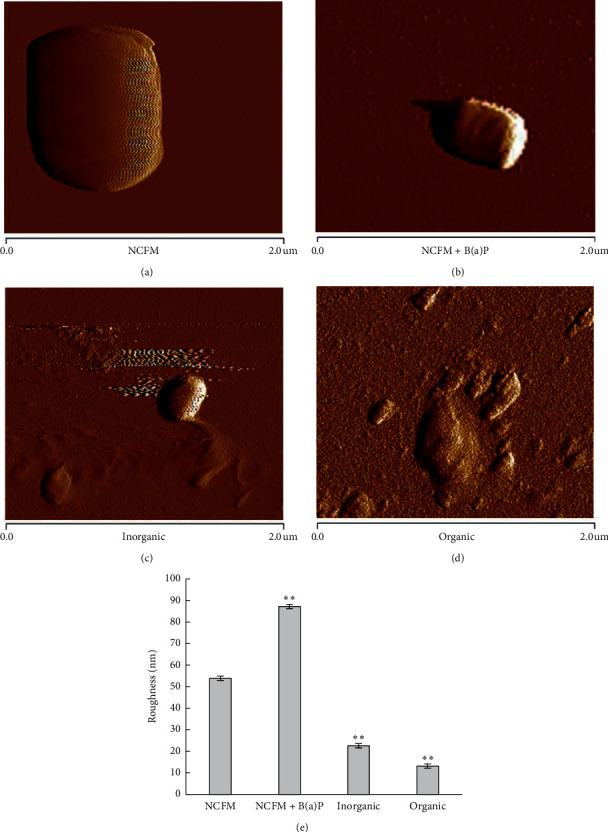
Factors affecting the binding ability of *L. acidophilus* NCFM to B(a)P existing in PM_2.5_, i.e., inorganic system and organic system: (a) AFM images of strain NCFM, (b) AFM images of B(a)P binding by strain NCFM, (c) AFM images of the inorganic system, (d) AFM images of the organic system, and (e) surface roughness analysis using AFM images (a, b, c, and d). Note: ^*∗*^*P* < 0.05 and ^*∗∗*^*P* < 0.01 compared with strain NCFM.

The morphology of the cells was measured by the AFM contact mode, and the single-cell topography of the control group and the three experimental groups was obtained (Figure 3). The morphology of strain NCFM binding B(a)P in the simulated inorganic system or organic system was shrinkage and edge-damaged, whilst the cells of the control group were smooth and intact, showed as normal *Lactobacilli* cells (Figures 3(a)–3(d)). Moreover, a decrease in the surface roughness of binding cells was also observed for the organic or inorganic system (Figure 3(e)).

## 4. Discussion

Currently, public sectors take regulation actions to reduce emissions and prevent inhalation in order to cope with the PM_2.5_ problem [22], but there is still no good solution for mitigating the injuries of toxins on the human body. To date, various physical and chemical ways have been tried to reduce the damage of PM_2.5_ to the human body. As a new type of biological filter medium, microorganisms have been used in indoor and automobile air purifiers [9]. *Lactobacillus* has been evaluated for its ability to remove various toxins, but very little was found in the literature on the studies of *Lactobacillus* as the main biological filter medium [23]. Our present study is designed to use lactic acid bacteria strains to remove the toxicity from PM_2.5_ in terms of their B(a)P binding ability.

Our previous studies confirmed that *Lactobacillus plantarum* 121, *Leuconostoc mesenteroides* DM1-2, *Lactobacillus acidophilus* NCFM, *Lactobacillus paralimentarius* 412, and *Lactobacillus pentosus* ML32 had good adsorption effects on B(a)P [21]. In our present study, it is interesting to note that five tested bacteria exhibited some ability to bind B(a)P, and at least 36.70% B(a)P was absorbed by these lactic acid bacteria strains. Additionally, their B(a)P absorbing capacity is observed to be species-specific. Among the tested strains, *L. acidophilus* NCFM has the best ability to bind B(a)P (60.0%). As potential decontaminating agents of aflatoxin B1 (AFB1) and B(a)P, the removal ability of lactic acid bacteria to the mutagenic compounds differs from strain to strain in various medium surroundings. Our results are in accordance with what have been reported so far [14, 24].

Regarding the composition of PM_2.5_, it includes sulfate, nitrate, ammonium salts, carbonaceous particles, metal particles, minerals, and the polycyclic aromatic hydrocarbon (PAH) family. The current studies have implicated that B(a)P is one of the causative agents in colon cancer [25]. Epidemiological studies provide evidence for dietary intake of PAHs which is associated with colorectal cancer risk [26]. On the question of the mechanism of colon tumorigenesis as a result of B(a)P ingestion, B(a)P could lead to a differential induction of cytochrome P450 both in the liver and colon, and its ability of malignant transformation of colon epithelial cells has also been documented [27, 28]. *Lactobacillus* mostly colonized rats' colon and ileum and has shown promise in preventing colon carcinomas in rats [29]. In addition to bind B(a)P, *Lactobacillus kefir* strains were reported to interact with metal ions via their binding ability [30]. Similar studies showed that *Lactobacilli* strains had good ability to bind various chemicals in various simulated environments [16, 31]. Interestingly, these studies have confirmed that the B(a)P binding efficiency is species-specific and depends on *Lactobacillus* cell structures. Several reports have shown that the adsorption of mycotoxins to the cell wall of *Lactobacillus* was attributable to their surface properties and mainly to their hydrophobicity [32, 33]. In addition, literatures have revealed that the adsorption of toxins by microorganisms mainly depends on the specific chemical composition of the cell wall such as the peptidoglycan, teichoic acids, and teichuronic acids of Gram-positive bacteria [34–36]. Therefore, it seems that adsorption of *Lactobacillus* is dependent on the hydrophobicity and cell wall structure. A note of caution is due to the limited binding site on the cell wall since binding efficiency changes with different environments. Most components in PM_2.5_ could be absorbed by *Lactobacillus* cells, but previous studies have not reported how the organic and inorganic systems which resemble PM_2.5_ affect *Lactobacillus* strains to bind with B(a)P. Dutton et al. directly simplified the most complex component environments into two types, i.e., organic and inorganic systems [37]. The simplified systems easily allow us to evaluate the toxicity of PM_2.5_ supplemented with B(a)P. In our case, we used the organic and inorganic systems which were supplemented with B(a)P *in vitro* to simulate PM_2.5_. Then, the ability of the tested *L. acidophilus* NCFM to bind B(a)P was evaluated in PM_2.5_. It was seen that the tested *L. acidophilus* NCFM bound B(a)P, and its B(a)P binding percentages were high in the simulated organic and inorganic systems. Thus, it is presumed from our *in vitro* test that use of selected lactic acid bacterial strains as a biofilter agent should have potential in removing the toxicity of PM_2.5_.

Numerous studies state that the roles of lactic acid bacteria in absorbing various chemicals such as ochratoxin A and aflatoxins depend largely on parameters such as incubation time, cultivation temperature, pH values, and viable cell counts [31]. The stability of the aflatoxin B1-bacteria complex appears to be species-specific [31]. This study confirms that the binding percentage of the mycotoxin by *Lactobacillus* is associated with incubation time [38]. Piotrowska found the ability of three lactic acid bacteria species in removing ochratoxin A, and heating made higher toxin-binding ability [39]. Aflatoxin B1 bound by lactic acid bacteria was pH-dependent, and a similar finding was also reported by Serrano et al. [40, 41]. Bacterial concentration is the other factor that strongly affects the removal of PAHs [17]. Studies showed that removal of aflatoxin M1 was significantly affected by microbial concentration [42]. Thus, the maximum adsorption of lactic acid bacteria cells to benzopyrene should be optimized. In our case, the optimized conditions are as follows: incubation temperature of 37°C, incubation time of 8 hr, pH of 5, and 1 × 10^10^ cfu/mL for the inorganic system. In this case, 92.74% B(a)P is removed by *L. acidophilus* NCFM. For the organic system with pH of 6, *L. acidophilus* NCFM binds 73.00% B(a)P only within 10 min when this strain is cultured at 37°C with a cell concentration of 1 × 10^10^ cfu/mL. *In vivo*, pH of the colon is stable at around 6.8, and the imbalance of pH in tissues is directly linked to diseases such as cancer [43]. These results suggest that *Lactobacillus* acidophilus NCFMTM may potentially prevent colon cancer development, and *Lactobacillus acidophilus* NCFMTM significantly suppressed AOM-induced colon carcinogenesis in a dose-dependent manner [44]. This corresponds with our earlier observations, which showed that the optimized conditions were suitable for B(a)P binding in the colon.

The presence of PM_2.5_ caused the B(a)P-binding cells to be changed in their morphology, as observed in our study (Figure 3). A similar report indicated that PM_2.5_ treatment destroyed the integrity of bacterial cells which shrank or suffered defects [15]. Importantly, the cell wall-prone status decreased because the main component of the cellular wall is composed of peptidoglycans [15]. This also accords with our earlier observations, which showed that more compounds existing in the complex PM_2.5_ were bound by NCFM strain cells compared to B(a)P environment only. Thus, analysis of AFM further proved that our selected NCFM strain was possible to be used as a precautionary agent for the reduction of PM2.5 pollutants due to its B(a)P binding ability.

## 5. Conclusions

Five tested lactic acid bacteria strains showed ability to bind B(a)P to some certain extent, but *L. acidophilus* NCFM exhibited the best capacity to bind B(a)P. At least 60% B(a)P was bound by this probiotic bacterium. This process of *L. acidophilus* NCFM to bind B(a)P was affected by incubation time, cultivation temperature, pH, and cell concentration. The simplification of PM_2.5_ into inorganic and organic systems provided a model for us to evaluate the roles of lactic acid bacteria strains in reducing the damage of the polluted environments to human beings. There were significant differences in the binding percentage of *L. acidophilus* NCFM to B(a)P between the two simulated systems, but our *in vitro* data indicated that strain NCFM exhibited good possibility to remove B(a)P existing in simulated PM_2.5_. Currently, environmental pollution situation becomes more and more serious, and thus, using probiotic bacteria to relieve environmental hazards will be a new attempt.

## Data Availability

The data used to support the findings of this study are included within the article.
